# Effect of Liuzijue exercise in different periods on circadian rhythm of blood pressure in patients with essential hypertension: A randomized trial

**DOI:** 10.1097/MD.0000000000036481

**Published:** 2023-12-01

**Authors:** Yixiao Chen, Nannan Liu, Yuyan Guo, Caiping Zheng, Dijun Fu, Yugang Cai, Kaidi Nie, Lina Xia

**Affiliations:** a School of Health Preservation and Rehabilitation, Chengdu University of TCM, Chengdu, China; b People’s Republic of China – Key Laboratory of Traditional Chinese Medicine Regimen and Health Industry Development, State Administration of TCM, Sichuan, China; c Key Laboratory of Traditional Chinese Medicine Health Preservation and Wellness in Sichuan Province, Sichuan, China; d Chongqing Bishan District Medical and Health Affairs Center, Chongqin, China; e Jin Cheng People Hospital, Jincheng, China; f The Southwest Hospital of AMU, Chongqin, China; g The Daying Hospital of Traditional Chinese Medicine in Suining City, Suning, China.

**Keywords:** blood pressure, circadian rhythm, essential hypertension, Liuzijue exercise

## Abstract

**Background:**

Exercising at different times of the day is a widely employed strategy for treating essential hypertension, aimed at enhancing the circadian rhythm of blood pressure. This study aimed to investigate the effects of Liuzijue exercise in the morning and afternoon on the circadian rhythm of blood pressure in patients with essential hypertension.

**Methods:**

This clinical randomized trial recruited 36 patients. They were randomly divided into 3 groups: morning exercise, afternoon exercise, and waiting treatment group. Randomization was performed at a ratio of 1:1:1, ensuring an equal distribution of participants among the 3 groups. Based on maintaining routine work and rest and continuing the original drug treatment, the exercise performed Liuzijue exercise for 12 weeks. The exercise time was 9:00 to 10:00 in the morning exercise group and 14:00 to 15:00 in the afternoon exercise group. The waiting treatment group did not perform any form of fitness exercise. The subjects in the 3 groups were monitored by 24-hour ambulatory blood pressure on the day before and after the first day.

**Results:**

After the 12-week Liuzijue exercise intervention, mean systolic blood pressure during the night significantly decreased in the morning exercise group (*P* < .01). In contrast, the waiting group experienced substantial increases in 24-hour mean systolic blood pressure (24hSBP), 24-hour mean diastolic blood pressure (24hDBP), mean systolic blood pressure during the daytime (dSBP), and mean diastolic blood pressure during the daytime (dDBP) (*P* < .01). Further analysis showed that the morning exercise group had significantly lower 24hSBP, 24hDBP, dSBP, dDBP, and mean systolic blood pressure during the night than the waiting group (*P* < .05). Additionally, the morning exercise group had lower 24hSBP and dSBP levels than the afternoon exercise group (*P* < .05). In contrast, the afternoon exercise group had lower 24hDBP and dDBP than the waiting treatment group (*P* < .05).

**Conclusions:**

The 12-week Liuzijue exercise in the morning regimen demonstrated superior efficacy in reducing 24-hour ambulatory blood pressure levels among patients with essential hypertension. Moreover, it facilitates the transition of non-dipper blood pressure patterns to dippers, thereby rectifying aberrant circadian rhythms.

## 1. Introduction

Essential hypertension is a chronic cardiovascular disease characterized by increased systemic arterial systolic and diastolic blood pressure. The abnormal increase in blood pressure usually leads to pathological blood pressure variation, and blood pressure which increases sharply or decreases excessively.^[1]^ Compared to the normal blood pressure rhythm of higher levels during the day and lower levels at night, individuals with essential hypertension exhibit an inability of their blood pressure to drop adequately during the night. This phenomenon can lead to an abnormal blood pressure state, characterized by elevated levels at night and lower levels during the day. Such abnormal patterns worsen damage to target organs and increase the risk of developing atherosclerosis, stroke, and other related diseases.^[2,3]^ Therefore, selecting the optimal timing for administering antihypertensive medications or implementing non-pharmacological therapies based on the fluctuation rhythm of blood pressure represents the principal intervention for effectively managing abnormal blood pressure patterns in individuals diagnosed with essential hypertension.^[4]^

Liuzijue exercise is a low-intensity aerobic exercise that combines resistance exercise with flexibility training.^[5]^ This exercise has been shown to effectively lower and regulate blood pressure variability in individuals diagnosed with essential hypertension. Its unique combination of exercises targets aerobic capacity and flexibility, yielding notable benefits for hypertension patients regarding blood pressure management and stability.^[6]^ However, few studies have investigated the combined effects of chronotherapy and exercise therapy, particularly in determining the optimal timing for the effectiveness of Liuzijue exercise in reducing blood pressure and normalizing the circadian rhythm of blood pressure. Therefore, this study aimed to investigate the effects of Liuzijue exercise in the morning and afternoon on 24-hour ambulatory blood pressure and circadian rhythm in patients with essential hypertension.

## 2. Methods

### 2.1. Trial design and oversight

This was a randomized controlled study to determine the effect of Liuzijue exercise in different periods on the circadian rhythm of blood pressure in patients with essential hypertension. The Medical Ethics Committee of the Affiliated Hospital of Chengdu University of TCM approved the study(ethical review number: 2019KL-047). Prior to commencing the intervention, a comprehensive explanation outlining the precise objectives, methodology, intervention schedule, anticipated advantages, and potential risks of the study was provided to all participants. By rigorously adhering to the principle of voluntary participation, individuals were duly informed, and their consent was obtained through the signing of an informed consent form prior to their inclusion in the study. To ensure the safety of the participants, stringent termination criteria were applied and adhered to throughout the study. Throughout the intervention period, the participants retained their autonomy to withdraw from the study based on their individual circumstances. Participants’ personal information and related records were kept strictly confidential.

### 2.2. Participants

From March 2019 to September 2020, patients diagnosed with essential hypertension who met the predetermined inclusion criteria were prospectively selected from the Ninth People’s Hospital of Qingyang District, Chengdu, and the Daying Hospital of TCM in Suining City, Sichuan.

Participants were included if they satisfied the following criteria: they were aged 50 to 80 years old; they met the diagnostic criteria of hypertension in the 2018 Chinese guidelines for the management of hypertension, hypertension was defined as systolic blood pressure ≥ 140 mm Hg (1 mm Hg = 0.133 kPa) and diastolic blood pressure ≥ 90 mm Hg^[7]^; they did not have systematic exercise habits, which referred to engaging in a singular exercise session lasting <30 minutes per day or participating in exercise fewer than 2 times per week; they were undergoing treatment with calcium channel blockers (such as nifedipine) had not altered their medication regimen in the immediate period prior to the study; they did not have any underlying conditions that impact motor function or exhibit severe motor dysfunction; they abstained from the use of any blood-activating and stasis-resolving traditional Chinese medicines, antibiotics, corticosteroids, or microbial preparations in the month preceding the study; they had no previous history of gastrointestinal disorders; they had not taken part in clinical trials involving Taijiquan, Baduanjin, or other traditional Chinese medicine exercise therapies within the preceding 6 months were included in the study.

Participants were excluded from the study if they met any of the following criteria: they had secondary hypertension; they had grade 3 hypertension, which is characterized by a systolic blood pressure equal to or >180 mm Hg and a diastolic blood pressure equal to or >110 mm Hg; they had hypertension that placed them at a high or very high cardiovascular risk level; they had hepatorenal and cardiac dysfunction, diabetes, chronic gastrointestinal diseases, or other serious complications; they were addicted to smoking and drinking for a long time; they had a history of severe trauma or surgery within the past 6 months and were deemed unsuitable for participation in sports activities; and they had a mental illness or poor compliance, referring to the inability or unwillingness to follow instructions or protocols.

Ultimately, 36 participants met the specified criteria. Professional statisticians employed the random number table method to allocate all subjects into the morning exercise group, afternoon exercise group, and waiting treatment group in a 1:1:1 ratio.

Due to the open and observational nature of the study, the blinding method was not employed. Nevertheless, efforts were made to utilize objective evaluation indicators to minimize human interference with the test results. Throughout the experiment, there was no communication among the 3 groups of subjects during the intervention, and the staff responsible for recruiting subjects had no knowledge of the experimental group.

### 2.3. Intervention

#### 2.3.1. Waiting treatment group.

Participants avoid participating in fitness exercises while following their current treatment program. Those currently taking medication should continue taking levamlodipine 2.5 mg once daily in the morning. Those not presently taking medication would not receive any additional medication. Participants were instructed to maintain a balanced diet, regular daily routine, stable mental state, and to avoid emotional triggers. Additionally, it was advised throughout the experiment to minimize antibiotics and traditional Chinese medicines that could affect the test indices, such as those promoting blood circulation or removing blood stasis. Following the 12-week intervention period, subjects in the control group were free to decide whether to incorporate Liuzijue exercise into their routine based on their personal preferences.

#### 2.3.2. Morning exercise group.

The participants in the treatment group adhered to their routine drug treatment program while maintaining the same diet and daily regimen as those in the waiting treatment group. Additionally, they performed Liuzijue exercises for 12 weeks. Before the formal commencement of the experiment, qualified healthcare professionals provided group instruction and training sessions on the “Liuzijue Exercise of Fitness Qigong.” The State Administration of Sports of China developed and endorsed the exercise in 2003. The teaching sessions covered essential components such as breath guidance, pronunciation for exhalation, key body movement points, and precautions. The aim was to ensure consistent execution of movements, posture, frequency, and mental focus among subjects. The regimen was officially implemented only after healthcare professionals confirmed that the issues had mastered the principles and steps of the Liuzijue exercise program. Exercise sessions were scheduled for 1 hour, specifically on Mondays, Wednesdays, and Fridays, from 9:00 to 10:00 each week. The exercise routine consisted of 2 minutes of preparatory activities, including chest expansion exercises, stretching exercises, and other actions to stretch out the joints and muscles of the whole body. This was followed by 56 minutes of exercise, with each set of movements lasting approximately 13 minutes. The entire collection of moves was performed 4 times, with a 2-minute break between each 2 times. After the exercise, there were approximately 2 minutes for finishing activities, where some simple relaxation movements and stretching, such as patting the leg muscles and moving all joints, were performed. Tailored exercise intensity was developed based on the individual circumstances of each participant. In other words, during the exercise process, the participant’s heart rate needed to reach the target heart rate for 5 to 10 minutes. The formula for calculating the target heart rate was: [(220-age)-resting heart rate]*(60–80%) + resting heart rate.

#### 2.3.3. Afternoon exercise group.

The sessions lasted for 1 hour between 14:00 and 15:00. The remaining interventions were consistent with the aforementioned details.

### 2.4. Outcome measures

The outcome measures were 24-hour ambulatory blood pressure and nocturnal blood pressure drop patterns. These measurements were taken on the day before and after intervention.

### 2.5. 24-hour ambulatory blood pressure

An ambulatory ECG blood pressure recorder (model BOX) manufactured by Zhongjian Instrument Co., Ltd in Wuxi City, Jiangsu Province, was used to monitor the following parameters: 24-hour mean systolic blood pressure (24hSBP), 24-hour mean diastolic blood pressure (24hDBP), mean systolic blood pressure during the daytime (dSBP), mean diastolic blood pressure during the daytime (dDBP), mean systolic blood pressure during the night (nSBP), and mean diastolic blood pressure during the night. These measurements were taken the day before and after the 12-week intervention period. Monitoring commenced at 8:30 and concluded at 8:30 the following day. For daytime monitoring (6:00–22:00), the ambulatory ECG and blood pressure recorder were configured to record data every 30 minutes. The cuff was automatically inflated to measure the blood pressure during this period. During nighttime monitoring (22:00–6:00 the next day), blood pressure was measured every 60 minutes using the same automatic cuff inflation method. Patients were instructed to keep their hands still and rest their arms naturally on their sides during blood pressure measurements using an ambulatory blood pressure instrument. This protocol ensured a minimum of one blood pressure reading per hour throughout the monitoring period, with at least 20 readings during the day and 7 reading at night. Additionally, the study aimed to obtain adequate blood pressure readings constituting over 70% of the total monitoring time.

### 2.6. Nocturnal blood pressure drop pattern

This study calculated the nocturnal blood pressure reduction rate according to the subjects’ changes in dSBP and nSBP. The specific calculation method was as follows: nocturnal blood pressure reduction rate = (dSBP − nSBP)/dSBP. Patients with 10% ≤ nocturnal blood pressure reduction rate <20% were classified as having a dipper nocturnal blood pressure decline pattern; patients with nocturnal blood pressure decrease rate < 10% or nocturnal blood pressure decrease rate ≥ 20% were classified as having a non-dipper blood pressure pattern.

### 2.7. Statistical analysis

Statistical analysis was performed using SPSS version 26.0. The experimental data are presented as mean ± standard deviation (*x̄*±s). One-way analysis of variance was used for comparison between groups. The constituent ratios were compared using the χ^2^ test or Fisher exact probability method, depending on the specific circumstances. For within-group comparisons before and after the experiment, a paired-sample *t*-test was used to assess the statistical significance of observed changes. *P* < .05 was considered significant.

## 3. Results

### 3.1. Demographic characteristics

**Figure 1.**
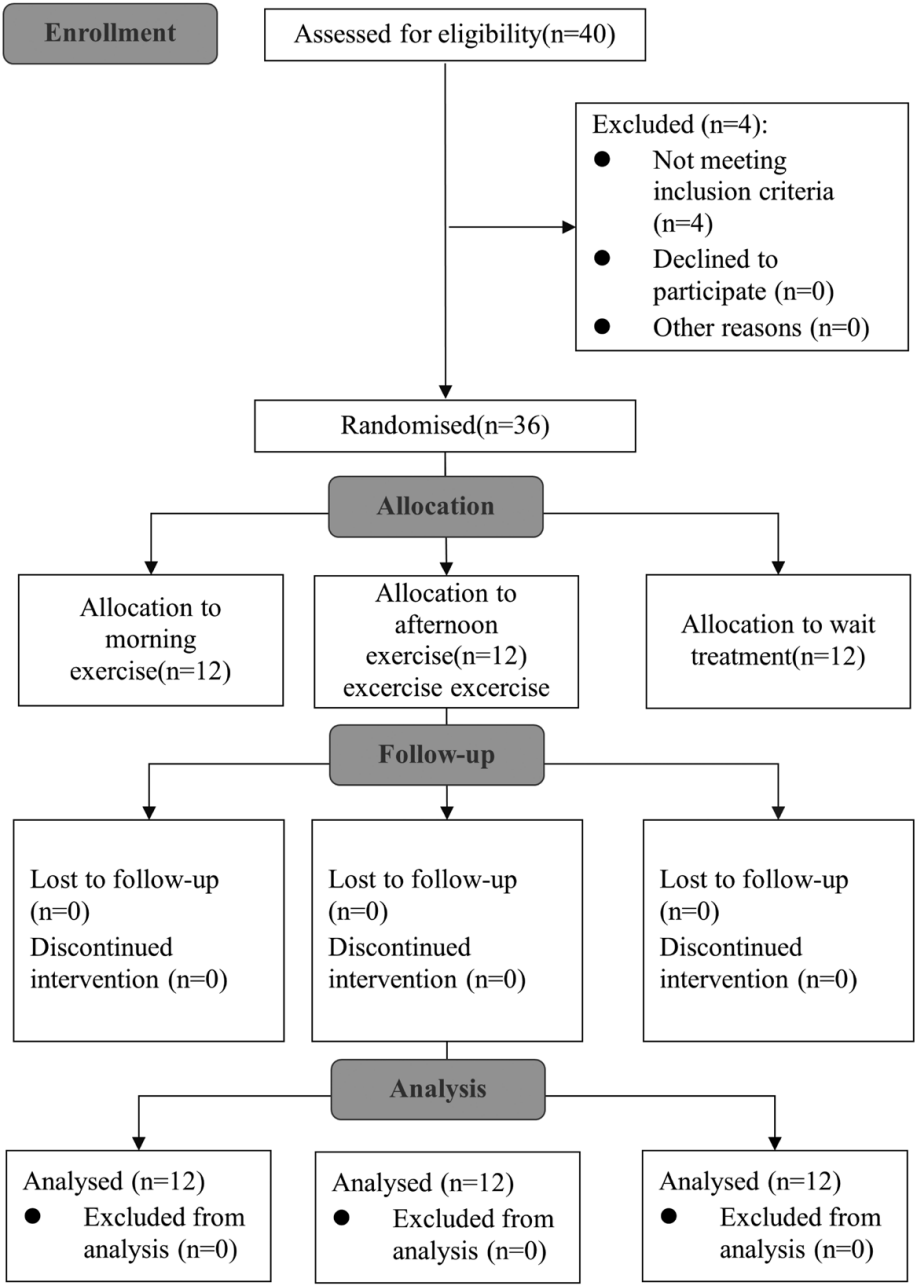
The workflow of the study.

Figure 1 illustrates the participant flow throughout the study, with 36 participants enrolled from the Ninth People’s Hospital of Qingyang District, Chengdu, and the Daying Hospital of TCM in Suining City, Sichuan between March 2019 and September 2020.

Patients in the morning exercise group comprised 8 male and 4 female patients, and the average age of these patients was 65.33 ± 4.66 years old (range:50–80 years old). In the afternoon exercise group, there were 6 male and 6 female patients, averaging 65.67 ± 5.97 years old. Patients in the waiting group comprised 5 male and 7 female patients, averaging 61.25 ± 4.39 years old. Other baseline characteristics include height, weight, and body mass index. Statistical analysis showed no significant differences in these characteristics among the 3 groups. Participants’ characteristics are presented in Table 1.

**Table 1:** Baseline characteristics.

	MEG (n = 12)	AEG (n = 12)	WTG (n = 12)	*P* value
Gender male/female	8/4	6/6	5/7	.458
Age(year)	65.33 ± 4.66	65.67 ± 5.97	61.25 ± 4.39	.073
Height (cm)	158.01 ± 8.26	156.33 ± 5.97	156.17 ± 4.95	.412
Weight (kg)	63.26 ± 6.56	58.08 ± 5.30	60.66 ± 6.69	.141
BMI (kg/m^2^)	25.38 ± 2.56	23.80 ± 2.20	24.87 ± 2.56	.332

AEG = afternoon exercise group, BMI = body mass index, MEG = morning exercise group, WTG = waiting treatment group.

### 3.2. 24-hour ambulatory blood pressure

**Figure 2.**
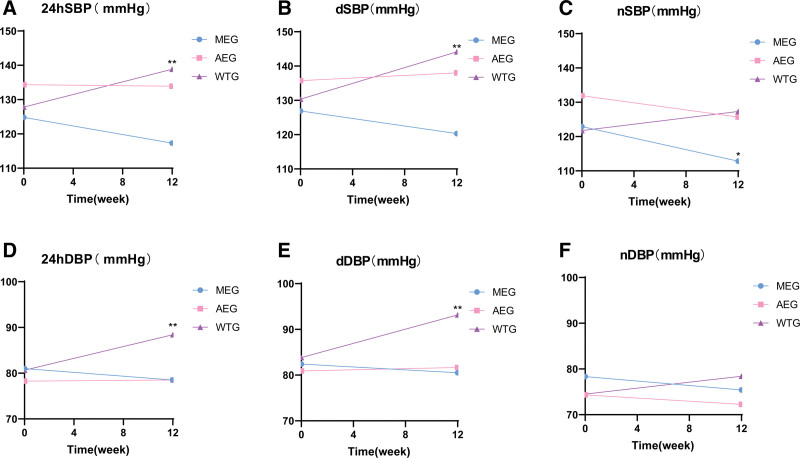
The changes of 24-hour ambulatory blood pressure in 3 groups of participants before and after the experiment. 24hDBP = 24-hour mean diastolic blood pressure, 24hSBP = 24-hour mean systolic blood pressure, AEG = afternoon exercise group, dSBP = mean systolic blood pressure during the daytime, dDBP = mean diastolic blood pressure during the daytime, MEG = morning exercise group. nSBP = mean systolic blood pressure during the night. nDBP = mean diastolic blood pressure during the night, WTG: waiting treatment group. ^*^P < .05 (compared with before the experiment). ^**^P < .01 (compared with before the experiment).

The results of 24-hour blood pressure changes in the 3 groups before and after the experiment are shown in Figure 2. Before the intervention, no significant differences were observed in 24-hour ambulatory blood pressure levels among the 3 groups (*P* > .05). After the 12-week Liuzijue exercise intervention, the nSBP in the morning exercise group decreased significantly. Conversely, the waiting group exhibited significant increases in 24hSBP, 24hDBP, dSBP, and dDBP (*P* < .01).

**Figure 3.**
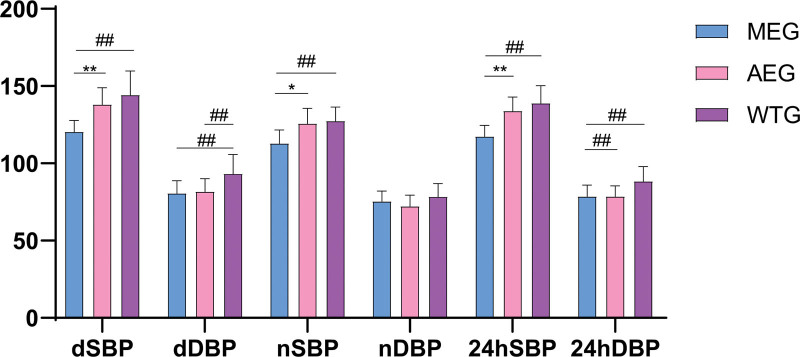
Comparison of 24-hour ambulatory blood pressure among the 3 groups after the experiment. 24hDBP = 24-hour mean diastolic blood pressure, 24hSBP = 24-hour mean systolic blood pressure, AEG = afternoon exercise group, dDBP = mean diastolic blood pressure during the daytime, dSBP = mean systolic blood pressure during the daytime, MEG = morning exercise group, nDBP = mean diastolic blood pressure during the night, nSBP = mean systolic blood pressure during the night, WTG = waiting treatment group. *P < .05 (compared with AEG). **P < .01 (compared with AEG). ##*P* < .01 (compared with WTG).

The difference of 24-hour ambulatory blood pressure among the 3 groups after the experiment is shown in Figure 3. The morning exercise group exhibited significantly lower 24hSBP, 24hDBP, dSBP, dDBP, and nSBP than the waiting treatment group (*P* < .01). Additionally, the 24hSBP, dSBP, and nSBP in the morning exercise group were lower than those in the afternoon exercise group(*P* < .05). Moreover, the afternoon exercise group displayed significantly lower 24hDBP and dDBP levels than the waiting group(*P* < .01). No statistically significant changes were observed in any of the other indices (*P* > .05).

### 3.3. Nocturnal blood pressure drop pattern

The number and proportion of patients with dipper blood pressure patterns before and after the trial in the 3 groups are shown in Table 2. There were no significant differences in the proportion of individuals with dipper blood pressure among the 3 groups before the intervention (*P* > .05). After completing a 12-week intervention involving Liuzijue exercise, notable improvements were observed in the participants’ abnormal nocturnal blood pressure drop pattern. In the morning exercise group, 2 subjects transitioned from a non-dipper nocturnal blood pressure drop pattern to a typical dipper nocturnal blood pressure decrease pattern, increasing the proportion of patients with dipper blood pressure from 16.7% to 33.3%. Similarly, 3 participants in the afternoon exercise group switched to the nocturnal blood pressure decrease pattern, leading to a proportional increase from 16.7% to 41.7%. There were no changes in the waiting group’ number or the proportion of patients with dipper blood pressure. Moreover, no significant differences were observed in the proportion of individuals with dipper blood pressure between the 3 groups before and after the experiment (*P* > .05).

**Table 2:** Comparison of the number (percentage) of patients with dipper blood pressure patterns between the 3 groups. patterns between the 3 groups.

	Before	After	*P* value
MEG	2 (16.7%)	4 (33.3%)	.64
AEG	2 (16.7%)	5 (41.7%)	.37
WTG	4 (33.3%)	4 (33.3%)	>.99
*P* value	>.99	>.99	

AEG = afternoon exercise group, MEG = morning exercise group, WTG = waiting treatment group.

## 4. Discussion

Human blood pressure exhibits a distinct circadian rhythm. It is influenced by various humoral factors, including the vascular endothelial system, renin–angiotensin–aldosterone system, catecholamines, other vasoactive substances, and autonomic and central nervous system.^[8,9]^ Circadian rhythm is characterized explicitly by a long-handle dipper curve, referred to as the “two-peak and one-valley” patterns. It begins with a gradual increase during the daytime and peaks at 9:00 to 11:00, after which it gradually declines. This pattern was followed by another rise around 15:00 to 18:00, followed by a gradual descent that persisted until nighttime. The abnormal increase in blood pressure in patients with essential hypertension is usually accompanied by weakening or disappearance of the blood pressure circadian rhythm. In contrast, high nocturnal blood pressure can transform patients from regular dipper nocturnal blood pressure patterns to non-dipper nocturnal blood pressure drop patterns. The latter increases the risk of stroke, renal dysfunction, and left ventricular hypertrophy and aggravates the damage to the cardiovascular system, kidney, and other organs.^[10]^ Therefore, some researchers have proposed the organic integration of hypertension treatment plans with the circadian rhythm of blood pressure fluctuation4. By adjusting the treatment timing, correcting the abnormal blood pressure circadian rhythm, and considering the antihypertensive effects, the objective is to attenuate target organ damage and effectively prevent and treat associated diseases.^[11]^

Liuzijue exercise is a traditional Chinese fitness practice emphasizing controlled exhalation and inhalation techniques, accompanied by pronouncing 6 specific characters: “Xu, He, Hu, Si, Chui, Xi.” These exercises were supplemented with simple arm-and-foot exercises to achieve the best results. Through exhalation and inhalation, the airflow vibrates and, exercises the 6 viscera of the liver, heart, spleen, lung, kidney, and gallbladder. The vibrations generated by the airflow were transmitted inside and outside the body simultaneously. The pronunciation of the corresponding mouth shape will resonates with the corresponding organs when transmitted internally. The guiding actions of the hands and feet jointly stimulate the circulation of the corresponding meridians of the Zang organs and promote the movement of qi and blood in the meridians to achieve the effects of fueling and dispelling evil and treating diseases. Compared to aerobic endurance exercises, such as jogging and swimming, Liuzijue presents a more conservative and gentle exercise approach with a narrower intensity range. Moreover, its versatility allows for its practical use in various environments, making it particularly suitable for individuals with high blood pressure. Notably, several studies have demonstrated that engaging in Liuzijue exercise can enhance vascular function, regulate intestinal flora composition, reduce blood pressure levels and stabilize blood pressure variability.^[6,12]^

The results of this study showed that the 24-hour ambulatory blood pressure of the 2 groups of subjects who received 12 weeks of exercise intervention improved to varying degrees after the experiment. Simultaneously, a rising trend was observed in the proportion of patients displaying a dipper blood pressure pattern within both the morning and afternoon exercise groups when compared to preexperiment measurements. Among them, the patients who engaged in the morning Liuzijue exercise exhibited a notable improvement in 24-hour ambulatory blood pressure. The research findings demonstrate that practicing Liuzijue exercises in the morning confers significant benefits to patients with essential hypertension. These benefits include the effective reduction of 24-hour ambulatory blood pressure, regulation of blood pressure variability, and facilitation of the transition from a non-dipper blood pressure pattern to a regular dipper blood pressure pattern.

The vasoconstrictive factors endothelin (ET) and vasodilatory factor nitric oxide (NO) are crucial vasoactive substances that maintain basal tension in blood vessels. ET induces vascular smooth muscle contraction, thereby increasing blood pressure and augmenting peripheral vascular resistance. Conversely, NO can relax vascular smooth muscle cells and inhibit their proliferation, which actively regulates blood pressure and prevents excessive elevations. Moreover, studies have revealed the potential involvement of both NO and ET in regulating the circadian rhythm of blood pressure.^[13,14]^ Relevant studies have shown that Liuzijue exercise can upregulate plasma NO levels in patients with hypertension by enhancing the NO release pathway.^[12]^ Furthermore, it effectively reduces the levels of ET-1 in patients, subsequently diminishing its vasoconstrictive effect while improving blood pressure.^[15]^ Therefore, Liuzijue exercise can promote restoration of the normal circadian rhythm of blood pressure. Shao found that administering antihypertensive treatment prior to the peak of blood pressure yielded the most pronounced effect in increasing plasma NO levels.^[16]^ In this study, the exercise timing for patients in the morning exercise group was aligned with the daily morning peak (9:00–11:00) of blood pressure. Consequently, engaging in Liuzijue exercise in the morning has a more favorable impact on enhancing the circadian rhythm of blood pressure.

Alterations in the circadian rhythm of blood pressure are correlated with disturbances in autonomic function. Such disruptions primarily manifest as an imbalance between the sympathetic and the parasympathetic nervous systems. Under normal physiological conditions, the human body engages in increased mental and physical activity during the daytime, which incites sympathetic nerve arousal and subsequently drive elevated blood pressure. Conversely, activity levels subside during nocturnal sleep, triggering heightened parasympathetic nerve activity and diminishing sympathetic nerve activity. Consequently, blood pressure decreases during nighttime hours.^[17]^ Erdem observed that the heart rate variability index was significantly lower in non-dipper-type hypertensive patients, suggesting that cardiac autonomic dysfunction is closely related to blood pressure rhythm changes in patients with early hypertension.^[18]^ Salles also found that the heart rate variability index decreased in patients with resistant hypertension, indicating that the weakened nighttime blood pressure drop was mainly related to significantly enhanced sympathetic nerve activity.^[19]^ A previous study demonstrated that regular aerobic exercise improved cardiovascular autonomic imbalance in individuals with hypertension.^[20]^ In our study, performing Liuzijue exercises in the morning significantly increased the daytime blood pressure. This improvement can be attributed to exercise’s ability to regulate autonomic nerve function and mitigate sympathetic nerve activity throughout the day.

Furthermore, the aberrant circadian rhythm of blood pressure is intricately linked to hemorrheology. Qin conducted a comparative analysis of the hemorheological parameters among 148 patients diagnosed with essential hypertension.^[21]^ The fibrinogen, Hct, ηb, ηp, ηbI, and erythrocyte agglutination index were significantly higher in the non-dipper group than in the dipper group. This phenomenon arises from the correlation between heightened blood viscosity and reduced vascular shear stress, resulting in diminished release of NO. These cascading effects compromise the vascular endothelial function, ultimately contributing to the development of abnormal circadian rhythms. Moreover, there is circadian rhythm in hemorheology. Wu found that the hemorheological parameters of rats demonstrated significantly higher blood viscosity during the day than night.^[22]^ A recent study demonstrated the effectiveness of Liuzijue exercise in reducing blood viscosity among individuals diagnosed with Diabetic Nephropathy.^[23]^ Hence, our study proposes a potential mechanism wherein engaging in Liuzijue exercise during the morning leads to an enhanced reduction in blood pressure viscosity throughout the day. Subsequently, it can lead to decreased blood pressure and improved abnormal blood pressure circadian rhythm in patients.

In the regulation of circadian rhythms in mammals, ambient light plays a highly significant role. Following the reception of external light cues, retinal ganglion cells convert ambient light intensity signals into nerve impulses.^[24]^ These impulses subsequently travel through the retinal hypothalamic pathway to reach the suprachiasmatic nucleus (SCN).^[25]^ The SCN, as the central component of the biological clock, houses oscillators that govern the circadian rhythms of various physiological activities in the human body.^[26]^ Hence, ambient light can modulate the circadian rhythms of these physiological processes by influencing the SCN. Gubin investigation demonstrated a notable increase in nSBP due to light exposure, resulting in an abnormal nocturnal blood pressure decline.^[27]^ Simultaneously, light hinders the secretion of melatonin (MT), subsequently leading to decreased blood pressure.^[28]^ Given that this study was conducted in an outdoor setting (in an open area outside the hospital), variations in ambient light levels may account for the divergent effects of Liuzijue exercise on blood pressure circadian rhythms in the morning and afternoon. Notably, afternoon light intensity is higher compared to the morning, which, under its influence, results in Liuzijue practitioners in the afternoon not exhibiting a significant improvement in 24-hour ambulatory blood pressure which, under its influence, results in Liuzijue practitioners in the afternoon not exhibiting a significant improvement in 24-hour ambulatory blood pressure.

In 1983, the World Health Organization recommended nondrug therapy as a preliminary and adjuvant treatment for mild-to-moderate hypertension. Previous studies have shown that Liuzijue exercise can effectively lower blood pressure and improve cardiovascular function. However, few studies have focused on the best performance of Liuzijue exercise. This study’s innovation combines chronotherapy with sports medicine, and find that the morning is more suitable for exercise with essential hypertension. In the future, we can further develop the influence of the duration and intensity of Liuzijue exercise on treating diseases and develop an exercise prescription that is more suitable for patients with essential hypertension.

### 4.1. Limitations

This study has several limitations. First, the sample size was small, with only 36 participants included, which may limit the generalizability of the findings. Second, the intervention duration was relatively short, consisting of only 12 weeks of Liuzijue exercise, which may need to be increased to capture the long-term effects or changes in the subjects’ blood pressure patterns. Additionally, the study only focused on the effects of Liuzijue exercise in the morning and afternoon on the circadian rhythm of blood pressure in patients with essential hypertension without exploring other potential impacts or variables.

It is noteworthy that in this study, 8 men were assigned to the MEG, whereas only 5 were assigned to the WTG. This variation can be attributed to the constraints imposed by experimental conditions and methodologies. Ben-Dov conducted ambulatory blood pressure monitoring in 3957 patients between 1991 and 2005, and his findings revealed that ambulatory blood pressure typically exhibited lower values in women compared to men.^[29]^ This physiological phenomenon may have exerted some influence on the outcomes of our study. Nevertheless, prior research has indicated that gender does not impact other circadian rhythm changes in blood pressure, specifically the transition between “dipper” and “non-dipper” patterns.^[30]^ Because the number of cases included in this study is small, and the ambulatory blood pressure of the subjects in the AEG group and the WTG group with fewer males is still higher than that in the MEG group after the experiment, we believe that the gender differences among the 3 groups do not bias the results of the study.

In future research, it would be beneficial to investigate the effects of exercise at different times of the day on vascular endothelial function, hemodynamics, or other relevant factors to provide a more comprehensive understanding.

## 5. Conclusion

In conclusion, this study revealed that morning Liuzijue exercise exhibited superior efficacy in enhancing abnormal blood pressure circadian rhythm among patients with essential hypertension, surpassing the effects of afternoon exercise. Furthermore, it facilitates the conversion of non-dippers to dippers. The specific mechanism of action warrants further exploration in future studies.

## Author contributions

**Conceptualization:** Kaidi Nie, Lina Xia.

**Data curation:** Yixiao Chen.

**Formal analysis:** Yixiao Chen.

**Investigation:** Yuyan Guo, Caiping Zheng, Dijun Fu, Yugang Cai.

**Methodology:** Yixiao Chen, Nannan Liu, Kaidi Nie, Lina Xia.

**Supervision:** Lina Xia.

**Visualization:** Yixiao Chen, Nannan Liu.

**Writing – original draft:** Yixiao Chen.

**Writing – review & editing:** Nannan Liu, Lina Xia.
